# Spasticity assessment based on the Hilbert–Huang transform marginal spectrum entropy and the root mean square of surface electromyography signals: a preliminary study

**DOI:** 10.1186/s12938-018-0460-1

**Published:** 2018-02-27

**Authors:** Baohua Hu, Xiufeng Zhang, Jingsong Mu, Ming Wu, Yong Wang

**Affiliations:** 1 School of Mechanical Engineering, Hefei University of Technology, No. 193 Tunxi Road, Hefei, 230009 China; 2 Department of Rehabilitation Medicine, Anhui Provincial Hospital, No. 1 Swan Lake Road, Hefei, 230001 China

**Keywords:** Spasticity assessment, sEMG, Onset detection, HHT marginal spectrum entropy, RMS

## Abstract

**Background:**

Most of the objective and quantitative methods proposed for spasticity measurement are not suitable for clinical application, and methods for surface electromyography (sEMG) signal processing are mainly limited to the time-domain. This study aims to quantify muscle activity in the time–frequency domain, and develop a practical clinical method for the objective and reliable evaluation of the spasticity based on the Hilbert–Huang transform marginal spectrum entropy (HMSEN) and the root mean square (RMS) of sEMG signals.

**Methods:**

Twenty-six stroke patients with elbow flexor spasticity participated in the study. The subjects were tested at sitting position with the upper limb stretched towards the ground. The HMSEN of the sEMG signals obtained from the biceps brachii was employed to facilitate the stretch reflex onset (SRO) detection. Then, the difference between the RMS of a fixed-length sEMG signal obtained after the SRO and the RMS of a baseline sEMG signal, denoted as the RMS difference (RMSD), was employed to evaluate the spasticity level. The relations between Modified Ashworth Scale (MAS) scores and RMSD were investigated by Ordinal Logistic Regression (OLR). Goodness-of-fit of the OLR was obtained with Hosmer–Lemeshow test.

**Results:**

The HMSEN based method can precisely detect the SRO, and the RMSD scores and the MAS scores were fairly well related (test: χ^2^ = 8.8060, *p* = 0.2669; retest: χ^2^ = 1.9094, *p* = 0.9647). The prediction accuracies were 85% (test) and 77% (retest) when using RMSD for predicting MAS scores. In addition, the test–retest reliability was high, with an interclass correlation coefficient of 0.914 and a standard error of measurement of 1.137. Bland–Altman plots also indicated a small bias.

**Conclusions:**

The proposed method is manually operated and easy to use, and the HMSEN based method is robust in detecting SRO in clinical settings. Hence, the method is applicable to clinical practice. The RMSD can assess spasticity in a quantitative way and provide greater resolution of spasticity levels compared to the MAS in clinical settings. These results demonstrate that the proposed method could be clinically more useful for the accurate and reliable assessment of spasticity and may be an alternative clinical measure to the MAS.

## Background

Spasticity is a serious and potentially disabling complication of stroke [1]. In the commonly accepted definition, spasticity is characterized by an increase in muscle tension during passive stretching, which is velocity-dependent and results from the hyperexcitability of the stretch reflex [2, 3]. Accurate and reliable assessment of spasticity is crucial for designing optimal treatment plans or evaluating potential effects of treatment interventions [4, 5].

At present, the dominant clinical method for assessing spasticity is the Modified Ashworth Scale (MAS), which provides for great simplicity in clinical assessment, but nonetheless represents a semi-quantitative method that depends on subjective physiotherapists evaluations based on experience [6–11]. Moreover, the method provides ambiguous descriptions such as “more marked (MAS 2), slight increase (MAS 1 and MAS 1+) in muscle tone”, which leads to equally ambiguous and imprecise results such as “1 to 1+” or “1+ to 2” [5].

The subjective and imprecise assessments of spasticity are not sensitive enough to slight changes of patient’s status [12]. They may misestimate the rehabilitation state of patients and lead to an inaccurate understanding of the characteristics of spasticity, thereby affecting the longitudinal follow-up of patients. They may also undermine the evaluation of the performance of therapeutic resources and misjudge the efficacy of an intervention in the clinical management of spasticity, thereby affecting the interventions, such as dose adjustment of anti-spasticity drugs or design of physiotherapy options.

Many studies have reported on the inaccurate and unreliable results obtained in the clinical assessment of spasticity, demonstrating the need to improve assessment accuracy and reliability [13–16]. However, most of these proposed methods have been criticized for being too cumbersome and inconvenient to apply in clinical settings [17–20].

Among the proposed methods, the tonic stretch reflex threshold (TSRT) seems to be the most promising approach [21]. The stretch reflex threshold (SRT) refers to the joint angle at which motoneurons and respective muscles begin to be recruited, and this onset of muscle recruitment is referred to as the stretch reflex onset (SRO), which can be detected based on surface electromyography (sEMG) activity [22, 23]. The TSRT represents the angle at which the SRO is detected when the muscle is at rest (i.e., at zero stretch velocity).

However, no consensus has yet been attained regarding the detection of the SRO [23]. In clinical settings, current approaches for detection of the SRO are mainly based on visual interpretation (VI) or time-domain signal analysis methods [24, 25]. These methods readily result in the erroneous detection of the SRO because sEMG signals are weak and vulnerable to interference. Additionally, the estimation of TSRT relies on a number of preliminary measurements with different stretching velocities that must be carefully undertaken in advance [26]. Finally, spasticity assessment based on the TSRT requires measurement of the joint angle and sEMG signal synchronously. It is quite difficult to realize ideal synchronous sampling in an actual testing environment because synchronization errors for separately sampled values always exist. In summary, spasticity assessment methods based on the TSRT are not practicable for clinical settings.

The nature of sEMG signals presents numerous processing challenges. sEMG signal is sparsely distributed in the time–frequency domain, and current detection approaches only expand signal in the time domain. The more detailed information in each frequency component is absent [25]. As such, a time–frequency representation is required to facilitate the SRO detection [25]. Moreover, sEMG signals seem to be nonlinear or even chaotic in nature. Therefore, nonlinear time series analysis methods, for example, entropy, may achieve improved performance over the methods relying on time domain parameter [27, 28]. Hence, a signal processing method that combines nonlinear dynamics and time–frequency analysis is more suitable for sEMG signal processing.

The Hilbert–Huang transform (HHT) is a relatively new time–frequency analysis approach, which is well suited for processing non-linear, non-stationary signals such as sEMG signals [29]. HHT marginal spectrum entropy (HMSEN) analysis is based on HHT and entropy, and the method of HMSEN has been used and acquired good performance in non-linear and non-stationary signal processing [30].

This study proposes a new practical clinical assessment method based on the HMSEN and the root mean square (RMS) of sEMG signals. The method detects the SRO based on the HMSEN of sEMG signals, and directly assesses spasticity quantitatively based on the difference between the RMS of a fixed-length sEMG signal obtained after the SRO and the RMS of a baseline sEMG signal.

The objectives of this study are to determine (1) the ability of the HMSEN to detect the SRO, (2) the relationship between the spasticity levels obtained clinically based on the MAS and the RMS difference measured from sEMG signals, (3) the test–retest reliability of the proposed method between days, and finally to (4) develop a clinical practical method of spasticity assessment, which is simple to operate and could provide greater resolution of spasticity level and better treatment and rehabilitation programs in clinical settings.

## Methods

### Subjects

Twenty-six patients (19 males and 7 females) with stroke participated in the study (mean age 53.85 ± 15.39, range 21–80 years). All the subjects were from Anhui Provincial Hospital. The study was approved by the ethics committee of Anhui Provincial Hospital. All subjects gave written informed consent approved by Anhui Provincial Hospital. For assuring confidentiality, the subjects were identified through the use of an Arabic numeral code, and no identifying information was recorded (i.e., no names, addresses, or contact information).

Subjects were included if they had (a) sustained a stroke; (b) elbow flexor spasticity; (c) at least a 90° passive range of motion in the elbow joint; (d) good awareness and no serious cognitive, visual, or auditory disorders.

Subjects were excluded if they had (a) other concurrent central nervous system disorders that may lead to myodystonia, e.g., Parkinson’s syndrome or multiple sclerosis; (b) other concurrent diseases that may compromise movement of the upper limb elbow joints, e.g., upper limb fracture or pain.

Each subject’s eligibility was carefully reviewed by an attending physician. The clinical characteristics of the 26 participants are listed in Table 1.

**Table 1:** Characteristics of the clinical test subjects

Subjects	Months after stroke	Age	Gender	Affected side	MAS score
S1	24	21	M	R	2
S2	1	64	M	L	1+
S3	24	48	F	R	1+
S4	2	76	M	R	1
S5	2	65	F	L	1
S6	0.5	63	M	R	1
S7	5	77	F	R	1
S8	13	30	M	R	1
S9	7	44	M	R	2
S10	2	62	M	L	1
S11	3	39	M	L	2
S12	38	80	M	L	1+
S13	1	52	M	R	1
S14	6	55	F	L	1
S15	24	38	M	R	1+
S16	0.5	64	M	L	1
S17	4	37	M	R	1
S18	1	54	M	L	1
S19	5	57	M	R	1+
S20	7	50	F	L	2
S21	2	48	F	R	2
S22	2	36	M	L	1+
S23	8	39	F	L	1+
S24	2	73	M	R	2
S25	6	61	M	L	1
S26	48	67	M	L	1+

*MAS* Modified Ashworth Scale, *M* male, *F* female, *R* right, *L* left

### Sensing system

Acquisition of the sEMG signal from the biceps brachii was accomplished using three electrodes via a three-point differential input. The patient’s skin at the points of electrode application was first wiped with an alcohol-soaked cotton swab to remove surface grease and dander. Two electrodes corresponding to the sEMG differential input were placed over the motor point of the biceps brachii muscles along the direction of the muscle fibers. The centers of the two electrodes were separated by 20 mm. The remaining electrode was designed as a reference point and was placed over the skin without any muscle activity.

The acquired sEMG signals were first filtered and amplified. The sEMG acquisition system consisted of a 10 Hz notch filter and a 500 Hz low-pass filter to remove the noise of the sEMG signals, and the filtered signals were amplified by a factor of 1000. The sEMG signals were then converted via a 12-bit analog to digital converter (ADC) (ADS1198, Texas Instruments, Dallas, TX, USA) at a sampling rate of 1 kHz, and transferred to a personal computer via USB ports. To store and analyze the data obtained during the study, data acquisition software were developed using Microsoft Foundation Classes (MFC). The trace of sEMG signals was continuously displayed on a monitor, and simultaneously stored on the computer. The data were employed to detect the SRO for each subject tested and investigate the correlation between the RMSD and MAS scores.

### Experimental protocol

**Fig. 1:**
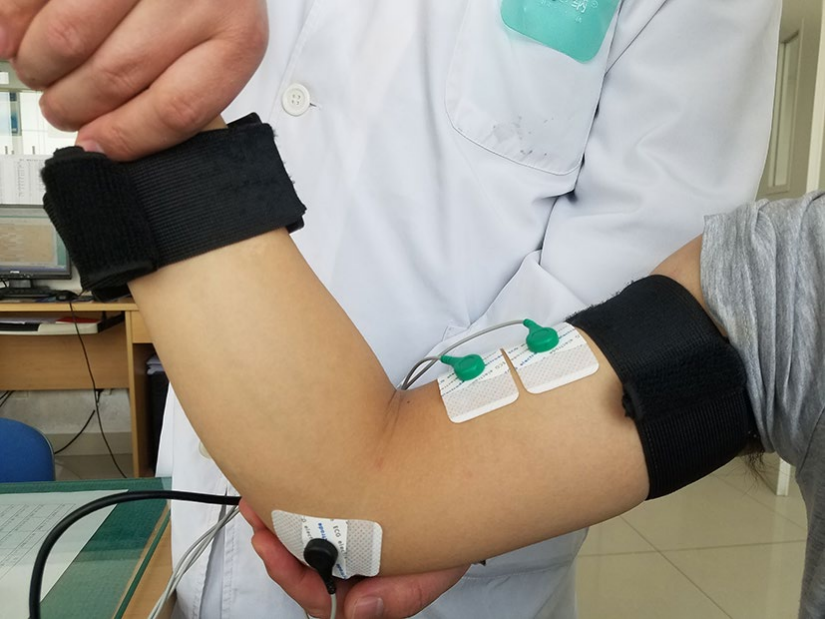
Clinical spasticity assessment based on MAS and sEMG signal acquisition

Each subject was tested in a seated position with the upper limb stretched toward the ground. Prior to assessment, a professional physiotherapist manually stretched the upper limb of each subject to assist the subject in adapting to the extension activity to avoid inaccuracy of spasticity assessment due to sudden extension. Subsequently, the physiotherapist performed a standard MAS scoring for each subject at a proper stretch velocity based on his professional experience. Here, we define elbow extension as a motion from full flexion, which represents the maximum flexion allowed by the upper arm and forearm without making contact between them, to full extension, representing the maximum extension allowed by the arm and the forearm. An MAS testing session consisted of a single elbow extension (Fig. 1). For purposes of statistical coding, ‘1+’ on the MAS was assigned a value of 1.5 in this study. Spasticity was evaluated in two sessions, denoted as test and retest, separated by an interval of 3–5 days. To minimize the influence of environmental and stress factors on spasticity assessment, all assessments were performed by the same physiotherapist, at approximately the same time of day, at the same location, and at a room temperature of 25 °C.

### SRO

Patients with spasticity have suffered damage to their upper motor neurons. The central lesion disrupts the balance of supraspinal inhibitory and excitatory inputs directed to the spinal cord, leading to a state of disinhibition of the stretch reflex. Decreased postsynaptic inhibition is involved in the hyperexcitability of the stretch reflex, which ultimately changes the electrophysiological output, manifesting as an increased complexity of sEMG signals in the time and frequency domain [31, 32]. This feature allows for the determination of the SRO using the HMSEN.

### HHT marginal spectral entropy

The fundamental component of HHT [29] is the empirical mode decomposition (EMD) method and the Hilbert transform. Application of EMD to an original time-based signal *x*(*t*) provides *n* intrinsic mode functions (IMFs) *c*_i_(*t*) and a residue signal *r*_*n*_(*t*) as follows:

1 $$ x(t) = \sum\limits_{i = 1}^{n} {c_{i} \left( t \right) + r_{n} \left( t \right)} . $$

For any *c*_*i*_(*t*), its Hilbert transform *H*(*c*_*i*_(*t*)) is defined as:

2 $$ H(c_{i} (t)) = \frac{1}{\pi }\int_{ - \infty }^{ + \infty } {\frac{{c_{i} (\tau )}}{t - \tau }} d\tau . $$

To construct the analytic signal:

3 $$ z_{i} (t) = c_{i} (t) + jH(c_{i} (t)) = a_{i} (t)e^{{j\varphi_{i} (t)}} , $$

where *a*_*i*_(*t*) and *φ*_*i*_(*t*) are the amplitude and instantaneous phase, which are respectively given as

4 $$ a_{i} (t) = \sqrt {c_{i}^{2} (t) + H^{2} [c_{i} (t)]} , $$

5 $$ \varphi_{i} (t) = \arctan \left( {\frac{{H[c_{i} (t)]}}{{c_{i} (t)}}} \right). $$

Correspondingly, *x*(*t*) can be expressed as

6 $$ x(t) = \text{Re} \sum\limits_{i = 0}^{n} {a_{i} (t)e^{{j2\pi \int {f_{i} dt} }} } , $$

where *f*_*i*_(*t*) is the instantaneous frequency, which is defined as

7 $$ f_{i} (t) = \frac{1}{2\pi }\frac{{d\varphi_{i} (t)}}{dt}. $$

The Hilbert–Huang spectrum is then defined to represent the frequency–time distribution of the amplitude:

8 $$ H(f,t) = \text{Re} \sum\limits_{i = 1}^{n} {a_{i} (t)e^{{j2\pi \int {f_{i} (t)dt} }} } . $$

Then, Eq. (8) is subjected to an integration over *t* to generate the Hilbert marginal spectrum of the signal:

9 $$ h(f) = \int_{ - \infty }^{ + \infty } {H(f,t)dt} . $$

The marginal spectrum offers a measure of total amplitude (or energy) contribution from each frequency value. Equation (9) can be rewritten to generate *h*(*i*) for discrete frequency points *f* = *i*Δ*f*:

10 $$ h(i) = \int_{o}^{T} {H(i,t)dt} ,\quad i = 1, 2, \ldots ,n, $$

where *n* represents the number of discrete frequency points within each analytic frequency band.

Correspondingly, the HMSEN is expressed according to the definition of information entropy as

11 $$ HMSEN = - \sum\limits_{i = 1}^{n} {p_{i} (\ln p_{i} )} ,\quad i = 1,{ 2}, \ldots ,n, $$

where *p*_*i*_ = *h*(*i*)/∑*h*(*i*), and *p*_*i*_ is the probability of occurrence of an event and here it refers the probability density of the spectrum and the occurrence probability of amplitude corresponding to the *i* th frequency.

The HMSEN values are then normalized in the range [0, 1], yielding the new HMSEN as follows:

12 $$ HMSEN = \frac{HMSEN}{\ln N}, $$

where *N* represents the sequence length of *h*(*i*).

### SRO detection algorithm

**Fig. 2:**
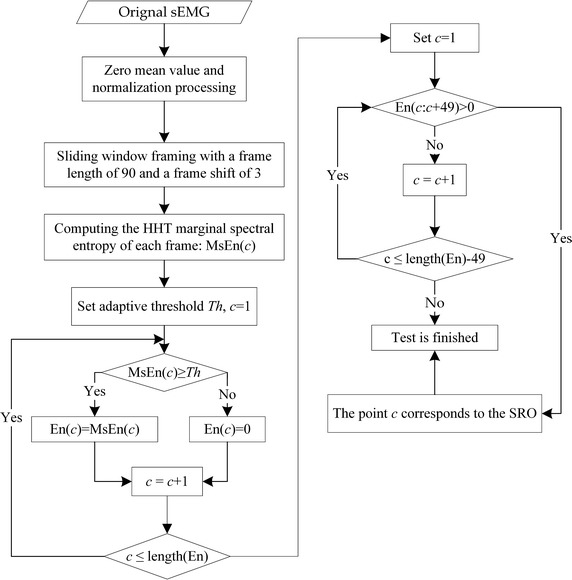
Algorithm flow chart for SRO detection

The HMSEN of sEMG signals obtained from the biceps brachii of patients was employed to detect the SRO according to the following procedure as shown in Fig. 2. First, the sEMG signal was framed by a sliding window of fix-length (*k* points) with a frame shift of *m* points. The HMSEN of each signal frame was calculated and denoted as *MsEn*. Subsequently, an adaptive threshold *Th* was set according to minimum and maximum values of *MsEn* [i.e., min(*MsEn*) and max(*MsEn*), respectively] as follows:

13 $$ Th = \hbox{min} (MsEn) + \lambda [\hbox{max} (MsEn) - \hbox{min} (MsEn)], $$

where *λ* is the sensitivity factor of *Th*. Here, the *MsEn* values were adjusted according to *Th*, where *MsEn* values less than *Th* were reassigned as 0, and *MsEn* values greater than *Th* were retained. The adjusted *MsEn* values are denoted as *En*. If, at a given point in time, an *En* value was greater than 0 and all *n* consecutive *En* values afterward were greater than 0, that point in time was determined to be the SRO. Considering a reasonable tradeoff between the computational burden and the desired level of accuracy, the values of these parameters were set as *k* = 90, *m* = 3, *n* = 50, and *λ* = 0.3 or 0.35.

The detection algorithm is outlined in Fig. 2, and was implemented in MATLAB (version 8.3, R2014a).

Activity bursts of short duration (25–40 ms) are considered to have little effect on the resulting kinetic output [33]. Therefore, the present study employed an interval [*t*_0_ − 50, *t*_0_ + 50] to obtain the recognition rate of the algorithm. The recognition rate is defined as

14 $$ Recognition\_rate = \frac{TD}{TD + FD}, $$

where *TD* and *FD* represent the number of true detection and false detection, respectively. A classification is counted as *TD* if the detected onset time is within the interval [*t*_0_ − 50, *t*_0_ + 50], and classifications are counted as *FD* if the detected onset time is outside the interval [*t*_0_ − 50, *t*_0_ + 50]. Here, *t*_0_ is the onset time obtained by the physiotherapist using VI.

The most cited method proposed by Calota A to measure TSRT [22], in which SRO was defined as the point at which the EMG signal increased 2 (standard deviation) SDs above the mean baseline EMG was selected for our performance comparison tests. Baseline EMG was the EMG activity while the subject was at rest before beginning the evaluation session. This method will be referred to as “SD detection” from now on.

The performance of the proposed method was evaluated using semi-synthetic sEMG signals where the onset time was artificially set. To construct the semi-synthetic sEMG signals employed for testing, we collected actual sEMG signals of the biceps brachii of elbow flexor spasticity patients, and two types of experimental signals were first identified. The first type represents quiescent baselines obtained during passive stretching. The second type represents clear sEMG signals where SRO occurred during passive stretching. The duration of each segment was 1000 ms.

### Spasticity assessment based on the RMSD

The SRO is accompanied by an excessive reflex activation of α-motoneurons, and the number of excitatory motoneurons and the electric activity are both increased. As a result, the RMS values of the sEMG signals increase with increasing muscle tension [34]. Hence, the spasticity level can be quantified using the RMS of the sEMG signals. However, some studies found that the sEMG signal showed differences in power spectrum even it was obtained when same action was performed by different individuals, and the differences still existed when the same individual performed the same action at different time [35]. Significant differences in signal amplitudes were also found in the baseline sEMG signals of the same subject. Hence, to eliminate the effects of individual differences of sEMG signals to the lowest, the difference between the RMS of sEMG signals obtained after the SRO and the RMS of baseline sEMG signals, henceforth defined as the RMSD, was employed to assess the spasticity level quantitatively. In consideration of the fact that the muscle continues contracting if the stretch is maintained (stretch velocity = 0) according to sEMG recordings [31], a fixed signal length of 1000 ms was adopted for assessing spasticity by this method. For short time-series, a fixed signal length of 500 ms was adopted.

The RMS is as follow:

15 $$ RMS = \sqrt {\frac{{\sum_{i = 0}^{N} {(X_{i} )^{2} } }}{N}} , $$

where *X*_*i*_ is the *i* th sampling value of sEMG signals.

### Statistical analysis

The relations between MAS scores and RMSD are investigated by Ordinal Logistic Regression (OLR) using R software. The quality of the RMSD in spasticity assessment was evaluated by how well the classes of the MAS test were predicted by the RMSD based on confusion matrix. Goodness-of-fit of the OLR was assessed with Hosmer–Lemeshow test with the R package *generalhoslem* [36]. The test–retest reliability was examined using (1) the interclass correlation coefficient (ICC) one-way random effects model for single measures [37], implemented using SPSS Statistics (IBM, Version 21.0), (2) Bland–Altman 95% limits of agreement (LOA) [38], implemented using MedCalc statistical software (version 15.8), and (3) standard errors of measurement (SEM) [39]. We employed ICC and SEM together because ICC is a relative measure of reliability that reflects the ability of the measurements to differentiate between participants, while SEM is an absolute measure. A one way ANOVA and post hoc test were used to compare the RMSDs among all subjects [20]. All statistical tests were conducted at a significance level of *p* = 0.05.

## Results

### SRO detection

**Fig. 3:**
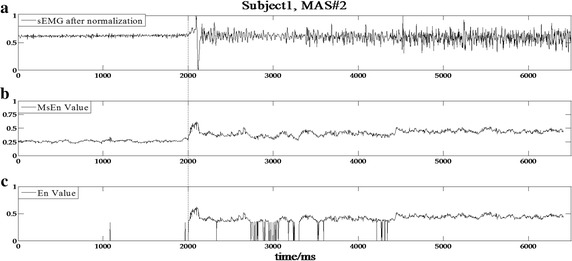
SRO detection result of clinical test subject 1. The vertical dashed line indicates the SRO

**Fig. 4:**
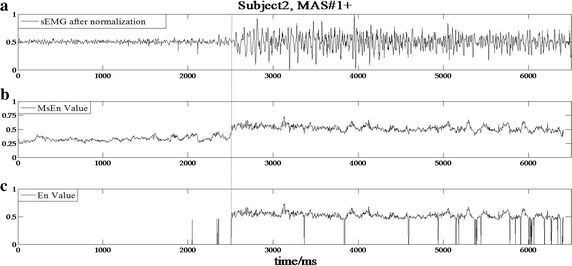
SRO detection result of clinical test subject 2. The vertical dashed line indicates the SRO

**Fig. 5:**
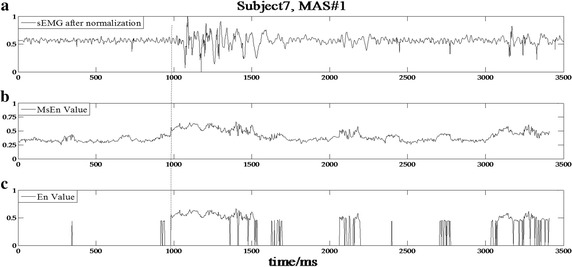
SRO detection result of clinical test subject 7. The vertical dashed line indicates the SRO

**Fig. 6:**
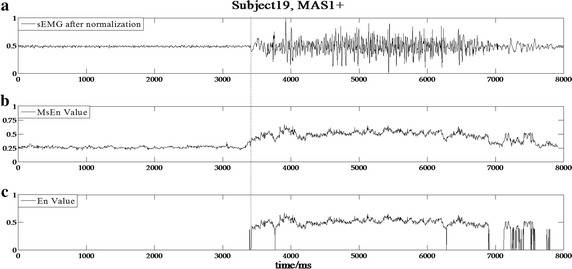
SRO detection result of clinical test subject 19. The vertical dashed line indicates the SRO

The data processing results based on the developed detection algorithm for subjects 1, 2, 7, and 19 with varying MAS levels are shown in Figs. 3, 4, 5 and 6, respectively. In the experimental results of subject 1 with an MAS score of 2 (Fig. 3), the HMSEN is approximately 0.25 prior to the SRO and approximately 0.5 after the SRO, which represents a significant difference before and after the SRO. Likewise, the experimental results of subject 2 with MAS 1+ (Fig. 4), subject 7 with MAS 1 (Fig. 5) and subject 19 with MAS 1+ (Fig. 6) demonstrate significant differences in their HMSEN values before and after the SRO as well, indicating that the HMSEN based method can reliably identify the SRO. According to Eq. (14), the results demonstrate that the HMSEN based method obtained a high SRO recognition rate of 96.3%.

### Performance comparison

**Fig. 7:**
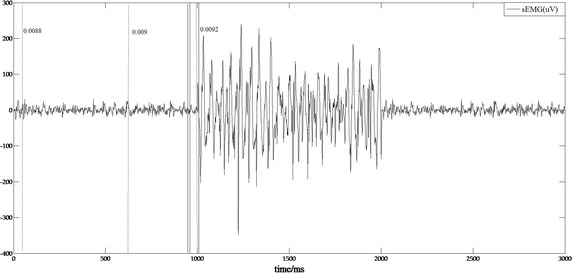
Performance comparison result: the vertical dashed line indicates SRO detected by SD detection, and the vertical solid line indicates SRO detected by our approach. The numbers next to the dashed line indicate the SD of the baseline sEMG signals. The solid lines from left to right indicate the SRO detection results when the sensitivity factor of *Th* is 0.3, 0.35 and 0.45

Figure 7 presents the performance comparison result. The vertical dashed lines indicate the SROs, which are detected by the SD detection method using different baselines. Visual inspection of the figure shows that a slight change in the baseline EMG signals may lead to a substantial change in the detection result (max error: 991 ms) when using SD detection. However, the SROs detected by the proposed approach, which are indicated by the vertical solid lines, show a smaller error (max error: 69 ms) and a better stability. Hence, the HMSEN based method appears to be more accurate and more robust than the SD detection method for SRO detection.

### Spasticity assessments

**Fig. 8:**
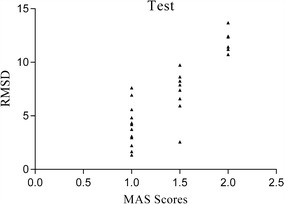
Scatterplots of RMSD vs MAS score (test)

**Fig. 9:**
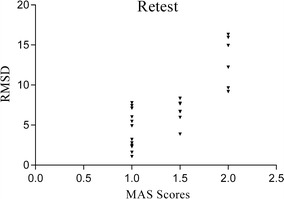
Scatterplots of RMSD vs MAS score (retest)

The RMSD of each subject is listed in Table 2. The scatter plot below shows the distribution of the RMSD of test (Fig. 8) and the RMSD of retest (Fig. 9) according to the MAS classification of the patients. The distribution of RMSD shows less variability than the RMSD of retest: except for three scores, the RMSD are monotonically related to the MAS classification. For the retest, the monotonic relation is less clear.

**Table 2:** Root mean square difference (RMSD) results for all clinical subjects

Subjects	RMSD (μV)		Subjects	RMSD (μV)		Subjects	RMSD (μV)	
	Test	Retset		Test	Retest		Test	Retest
S1	12.3626	14.9392	S10	3.0899	2.77	S19	7.4094	6.7106
S2	7.8972	5.9526	S11	11.4453	12.2285	S20	12.4408	16.3307
S3	6.6086	7.7197	S12	5.9376	7.6495	S21	10.7271	9.6359
S4	3.7376	2.3069	S13	1.3423	1.0797	S22	8.2567	8.347
S5	4.3297	4.9061	S14	4.8374	7.7962	S23	9.7447	6.6587
S6	7.6125	7.1067	S15	2.5677	3.8826	S24	11.2206	9.1761
S7	2.9684	3.2001	S16	4.1903	6.0266	S25	6.9481	7.4326
S8	1.6711	1.6053	S17	5.5998	5.4565	S26	8.6382	7.7777
S9	13.6893	15.9421	S18	2.2286	2.4608

An OLR was performed with the three levels of the MAS score as outcome measure and RMSD test as a predictor. The Hosmer–Lemeshow test (Table 3) shows significant goodness of fit and that the RMSD scores and the MAS scores are fairly well related (χ^2^ = 8.8060, *p* = 0.2669). Regarding the retest, the RMSD scores and the MAS scores are also fairly well related (χ^2^ = 1.9094, *p* = 0.9647), which demonstrates that the RMSD can quantitatively assess the level of upper limb spasticity.

**Table 3:** Goodness-of-fit of the OLR assessed with Hosmer–Lemeshow test

Test		Retest	
χ^2^	*p*	χ^2^	*p*
8.8060	0.2669	1.9094	0.9647

The confusion matrix (Table 4) shows how the MAS scores predicted with the RMSD (test) correspond to the observed MAS scores of the patients. The six patients who were classified with a MAS score of 2, all have a predicted score of 2 based on their RMSD scores. Not all patients are correctly classified: two patients were classified with MAS score of 1, but the predicted score based on RMSD is 1+, and for another two patients exactly the opposite occurs: observed MAS score is 1+, predicted based on RMSD is equal to 1. Overall the accuracy of the test is good: 0.85, 95% CI = (0.65, 0.96), which means that 85% of the patients were correctly classified. The confusion matrix of retest (Table 5) shows that the patients are less well classified based on the RMSD of retest: overall accuracy of the retest is equal to 0.77, 95% CI = (0.56, 0.91), which means that 78% of the patients were correctly classified.

**Table 4:** Confusion matrix (test)

	Actual		
	1	1+	2
Predicted			
1	10	2	0
1+	2	6	0
2	0	0	6

**Table 5:** Confusion matrix (retest)

	Actual		
	1	1+	2
Predicted			
1	9	2	0
1+	3	6	1
2	0	0	5

However, the goodness-of-fit of the OLR when considering only the RMS of sEMG signals after the SRO are (test: χ^2^ = 7.3070, *p* = 0.3976; retest: χ^2^ = 3.8367, *p* = 0.7984), and overall accuracy of the test and retest are 0.77 and 0.62 predicted predicting MAS scores with the RMSD respectively. These results mean the RMSD can achieve improved performance over the RMS in spasticity assessment, and decrease the individual difference effect to some degree.

### Test–retest reliability

**Fig. 10:**
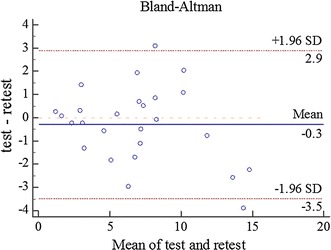
Bland–Altman plots with 95% limits of agreement (dashed lines). The X-axis shows the mean RMSD across the two assessments, and the Y-axis shows the difference in RMSD between the two assessments

The results of the test–retest reliability study are listed in Table 6. In addition, a Bland–Altman plot showing the mean RMSD over a period of two test days (X-axis), the difference in the RMSD values between the test days (Y-axis), and the 95% LOA (dashed lines), is presented in Fig. 10. We note from Table 6 that the ICC scores indicate good agreement between the two tests with an ICC of 0.914. The SEM value is very small (1.137). Finally, the Bland–Altman 95% LOA results also suggest a small bias with 92.3% (24/26) of the data points lying within the LOA.

**Table 6:** Test-retest reliability results

ICC			SEM	Bland–Altman LOA			
ICC	95% CI			D	SD	95% LOA	
0.914	0.819	0.960	1.137	− 0.29	1.63	− 3.5	2.9

### Spasticity level discrimination performance of the MAS

Through a post hoc analysis by means of the Bonferroni test, it was found that the differences between the RMSD values of the MAS 1 group and that of the MAS 1+ group (retest: *p* = 0.089) were not significant.

## Discussion

The objective of this study was to evaluate the level of spasticity easily, accurately, and reliable by combining the characteristics of both the MAS and sEMG signals. One advantage of the proposed approach is that the elbow flexors are manually stretched by the rater, instead of by isokinetic-dynamometer-like devices which may make the spasticity assessment more complicated. Another advantage is that it measures the RMS of sEMG signals after the SRO, rather than the TSRT, and the strict synchronous acquisition of the sEMG signals and the joint angle is no longer needed. Hence, the proposed method is more practical in clinical practice due to its compactness and portability. It is not clear whether it is absolutely necessary to apply constant velocity when measuring spastic joint stiffness [40]. Therefore, our manual methods and device might be a better measure of spasticity than those used in current practice and play an important role in further studies for spasticity measurement in clinical settings. Additionally, a novel approach for detecting SRO was presented based on time–frequency analysis and nonlinear dynamics, which is robust in detecting the SRO in clinical settings.

### Patient selection

Patients with MAS score 0, 3 and 4 are excluded from this experiment because the instructions of MAS score 0 and score 4 are obvious: either no resistance felt (score 0) or no movement possible (score 4) [41]. Therefore, the evaluation of spasticity levels of patients with MAS 0 and MAS 4 is simple and clear, and the chances of misdiagnosis barely exist. What’s more, the ROM of patients with MAS score 3 is very small, the chances of misdiagnosis barely exist as well. However, the descriptions of MAS score 1, 1+ and 2, such as “more marked (MAS 2), or slight increase (MAS 1 or 1+) in muscle tone”, as we discussed in Background, are qualitative and ambiguous Therefore, we only recruited patients with MAS score 1, 1+ and 2. Patients with these scores comprise the majority, and physiotherapists are tending to make mistakes when assessing the spasticity levels of patients with these scores in a clinical setting. Furthermore, the distribution of our dataset is similar to many studies [42–44], which indicate that only a small percentage of individuals with chronic stroke exhibit scores at the upper two levels of the MAS under passive extension of the elbow.

### Results analysis

We note that the recognition rate of 96.3% obtained by the HMSEN is considerably greater than the recognition rate of 87.5% correspondence with VI using the Teager-Kaiser Energy Operator method [23]. Hence, the HMSEN based method achieved improved performance over the methods relying on conventional SD detection and the Teager Kaiser Energy domain. The HMSEN also presents definite advantages relative to the sample entropy of sEMG signals, which currently has been employed for detecting the onset of muscle activity [45]. Despite the significant performance of the standard sample entropy in physiological signals processing, it does require more various parameters, such as the dimension and the tolerance, first be empirically established to obtain accurate results [46]. However, the HMSEN approach needs less parameter settings, resulting in enhanced practicability for clinicians. Overall, the HMSEN approach for SRO detection is more suitable for clinical settings.

While this study provides some evidence for the good test–retest reliability of the system, Rankin and Stokes [47] have recommended that a large sample size of at least 50 is required for an accurate evaluation of the 95% LOA. As such, our sample size of 26 subjects does not provide for an accurate calculation of the Bland–Altman LOA, which limits the credibility of our test–retest reliability study. Additionally, unlike the ICC, the SEM provides an absolute index of reliability [39], which is of greater usefulness to clinicians. In this study, the RMSD varies from 1.0797 to 16.3307, so an SEM of 1.137 may have little effect on the spasticity level measured by the RMSD. However, the SEM value obtained was slightly greater for patients with MAS 1 than that obtained for other spasticity levels, and we preliminarily hypothesize that this may be the result of the small sample size. Thus, better test–retest reliability data can be expected for a larger sample size.

No significant differences of the average RMSDs between the MAS1 and MAS1+ groups were detected (retest: *p* = 0.089). Hence, this supports the questionability of the MAS due to its subjectivity and the equivocal descriptions employed, where the rate of change in passive resistance and the joint angle at the onset of stretching are what physiotherapists actually examine based on their experience [48]. In this sense, spasticity assessment based on RMSD is aligned with the study of Damiano DL in [48]. Additionally, the MAS grades spasticity through only 6 levels, but the RMSD varies from 1.0797 to 16.3077. Therefore, our manual method might be more reasonable for providing an accurate, reliable, detailed, and discriminative evaluation of spasticity once its reliability and validity are established further.

### Study limitations

Considerable work is required before the proposed system can be adopted as a practical clinical tool. Firstly, we note that examiners typically did not take the velocity-dependent character of spasticity into account during MAS evaluation, and the elbow extension of the patients was conducted according to their experience and visual feedback. As such, the RMSD must also be considered at different stretch velocities. Thus, we plan to investigate the impact of the stretch velocity on the experimental results in a future study, as well as to develop a clinically simple method of eliminating the influence of different stretch velocities.

Secondly, we acknowledge that the subjects in this study all came from one rehabilitation centre and that the sample is thus relatively small. Therefore, this system should be applied to a larger number of spasticity patients with a greater variation in spasticity levels, particularly patients with MAS 1+ and 2 levels. Besides, we will need to apply our system to patients with various upper motor-neuron disorders, in order to demonstrate its feasibility.

Thirdly, spasticity level was determined by means of the MAS by one physiotherapist, following routine clinical test procedures. However, the MAS has been criticized for inaccuracy and unreliability [7], which may have impact on results. Hence, the inter-rater reliability should be considered in the future. In addition, the value of other clinical scales, for example, Modified Tardieu Scale, as an indicator for the level of spasticity should be further explored. Besides, the SRO detection results may be biased by the length of the sliding window and the interval which is set based on the onset time detected using VI. The assessment of spasticity levels may be biased by the length of sEMG signals for the calculation of RMSD. How to set the optimal parameters for more precise detection of the SRO and more accurate assessment for spasticity should be future studied.

A final limitation of this study is that we only use one single clinical parameter (RMSD) to assess the spasticity levels of patients. In a future study, we plan to equip our device with extra force sensors and use multiple parameters to assess spasticity level, such as the mean power–frequency of sEMG signal, the joint resistance and the SRT.

### Clinical implications

A quantitative method to measure spasticity may provide physiotherapists with accurate understanding of spasticity and optimal treatment plans for patients. The proposed method takes the advantages of the MAS and portable manual system and is easy to operate. Hence, the proposed method permits physiotherapists to assess the level of spasticity reliably, easily and unrestrictedly. Compared with the clinical scales for spasticity assessment, which are not sensitive enough to status changes of patients, it can provide clinicians with a greater resolution of spasticity level. Another advantage is that RMSD values can also provide clinicians with insight into the etiology of spasticity in the future, for it mainly demonstrates the neural response to the muscle stretch [49]. From a therapeutical point of view, the proposed method can provide better treatment program. For patients, the proposed method assesses their spasticity level manually, which avoid the uncomfortable muscle stretches, which are forcibly made by locking up the extremity in an instrument [20, 44]. From the view of rehabilitation, it is likely to derive a standardized clinical evaluation protocol for patients with different spasticity levels. It can provide a more refined classification of spasticity grades, which may lead to better healthcare services and better therapeutic intervention for patients, and facilitate the rehabilitation of patients in clinical practice.

## Conclusions

This report presented a novel framework for upper limb spasticity assessment based on the analysis of sEMG signals for detecting the SRO and for the direct quantitative assessment of spasticity. Experimental results demonstrated that the HMSEN based method could precisely detect the SRO. The statistical analysis results demonstrated a strong correlation between the RMSD and the MAS scores obtained for spasticity patients, and good test–retest reliability. This method is manually operated and easy to use, so it can make quantitative spasticity assessment readily available in clinical practice, and therefore enhancing diagnostic and recovery of patients. The preliminary results suggest that the proposed method could potentially provide useful clinical information, and may be viewed as an alternative clinical measure to the MAS in clinical settings.

## Abbreviations

sEMG: surface electromyography
HHT: Hilbert–Huang transform
HMSEN: Hilbert–Huang transform marginal spectrum entropy
RMS: root mean square
SRO: stretch reflex onset
RMSD: RMS difference
MAS: Modified Ashworth Scale
OLR: Ordinal Logistic Regression
TSRT: tonic stretch reflex threshold
SRT: stretch reflex threshold
VI: visual interpretation
M: male
F: female
R: right
L: left
ADC: analog to digital converter
MFC: Microsoft Foundation Classes
EMD: empirical mode decomposition
IMF: intrinsic mode functions
TD: true detection
FD: false detection
SD: standard deviation
ICC: interclass correlation coefficient
LOA: limits of agreement
SEM: standard errors of measurement
