# Correction to: Spasticity assessment based on the Hilbert–Huang transform marginal spectrum entropy and the root mean square of surface electromyography signals: a preliminary study

**DOI:** 10.1186/s12938-019-0642-5

**Published:** 2019-03-20

**Authors:** Baohua Hu, Xiufeng Zhang, Jingsong Mu, Ming Wu, Yong Wang

**Affiliations:** 1 School of Mechanical Engineering, Hefei University of Technology, No. 193 Tunxi Road, Hefei, 230009 China; 2 Department of Rehabilitation Medicine, Anhui Provincial Hospital, No. 1 Swan Lake Road, Hefei, 230001 China

## Correction to: Biomed Eng OnLine (2018) 17:27 10.1186/s12938-018-0460-1

After the publication of the original article work [1], it was highlighted that the circuit information and the A/D converter used for acquiring sEMG signal were not correctly reported in “Methods” section. The circuit included a 10 Hz high-pass filter instead of 10 Hz notch filter as stated in the original article. The A/D converter was ADS7818, instead of ADS1198 as stated in the original paper. The authors apologize to the readers for the inconvenience.
